# Impact of high dose radiotherapy for breast tumor in locoregionally uncontrolled stage IV breast cancer: a need for a risk-stratified approach

**DOI:** 10.1186/s13014-023-02357-7

**Published:** 2023-10-11

**Authors:** Nalee Kim, Haeyoung Kim, Won Park, Won Kyung Cho, Tae Gyu Kim, Young-Hyuck Im, Jin Seok Ahn, Yeon Hee Park, Ji-Yeon Kim

**Affiliations:** 1 Department of Radiation Oncology, Samsung Medical Center, Sungkyunkwan University School of Medicine, 81 Irwon-ro, Gangnam-gu, Seoul, 06351 Republic of Korea; 2 Department of Radiation Oncology, Samsung Changwon Hospital, Sungkyunkwan University School of Medicine, Changwon, Republic of Korea; 3 Department of Medicine, Samsung Medical Center, Sungkyunkwan University School of Medicine, Seoul, Republic of Korea

**Keywords:** Metastatic breast cancer, Palliative, Radiation therapy

## Abstract

**Aim:**

Patients with locoregionally uncontrolled breast tumors are frequently referred for breast palliative radiotherapy (PRT) to mitigate symptoms. We analyzed the outcomes following breast PRT to optimize PRT according to risk groups.

**Methods:**

We reviewed 133 patients who underwent breast PRT. A median total dose of 45 Gy was prescribed with an equivalent dose in 2 Gy fractions (EQD2, α/β = 3.5) of 53 Gy. The Cox proportional hazards model was used to analyze the prognostic factors of local control (LC).

**Results:**

Most (90.2%) had polymetastatic disease (> 5 lesions), and 48.9% had bone metastasis. With a median follow-up of 17.2 months, the 2-year LC and overall survival (OS) rates were 49.4%, and 48.3%, respectively. Multivariable analyses demonstrated progressive or mixed responses outside the breast and > 2 lines of previous therapy as adverse features for clinical outcomes. Group 1 (0 risk factors) showed favorable 2-year LC and OS of 63.9%, and 72.8%, respectively, whereas group 3 (2 risk factors) showed the worst outcomes of 0%, and 6.8%, respectively. Breast PRT with EQD2 ≥ 63 Gy showed a significant benefit in LC for group 1 and marginal benefit (*p* = 0.055) for group 2, but no improvement for group 3 (*p* = 0.300).

**Conclusion:**

Breast PRT showed favorable LC outcomes in patients with stable disease outside the breast and treated with ≤ 2 lines of systemic treatment. Our findings warrant future clinical trials investigating the role of higher than palliative dose and early intervention of PRT in stage IV patients.

**Supplementary Information:**

The online version contains supplementary material available at 10.1186/s13014-023-02357-7.

## Introduction

Recent advances in systemic treatments, which have improved overall outcomes in stage IV breast cancer with uncontrolled primary tumors have motivated physicians to integrate locoregional treatment in these patients [1]. Historically, radiotherapy (RT) or surgery was conceived as for the palliation of symptomatic local disease [2–4]. Meanwhile, for some primary diseases (e.g. lung and prostate cancer), local treatment of the intact primary tumor has been hypothesized to improve outcomes in stage IV disease [5–8]. In this context, several studies have been conducted to evaluate the therapeutic role of local treatment, including surgery and/or postoperative RT for primary breast tumors in de novo stage IV disease [9–19]. Although prospective trials failed to demonstrate survival benefit, most studies showed benefit in local control[16–19]. In addition, recent prospective randomized trials investigated the metastasis-directed local therapy in oligometastatic and oligoprogressive metastatic breast cancer, but early results of these trials failed to show survival benefit [20, 21].

In patients with unresectable breast tumors, polymetastatic status, or recurrent stage IV breast cancer, breast palliative RT (PRT) is administered with the aim of alleviating breast pain, bleeding, and discharge symptoms [22]. In addition, following the development of modern and effective systemic agents, breast PRT is also considered when persistent or progressive breast tumors are detected during systemic treatment in stage IV disease with the expectation of local disease control. However, there is limited literature on the oncologic impact of breast PRT in addition to its ability to relieve symptoms from unresectable breast tumors. Moreover, it remains unclear which breast PRT regimen is optimal for achieving tumor control while minimizing the treatment burden. Therefore, we evaluated the treatment outcomes of PRT in patients with uncontrolled breast tumors. Through this analysis, we aimed to build a therapeutic strategy in which the breast PRT regimen was adjusted according to the risk groups.

## Materials and methods

### Patient population

After receiving approval from the institutional review board (SMC 2023–01-030), we retrospectively reviewed 169 patients who underwent PRT for locoregionally uncontrolled breast cancer between January 2010 and June 2021 at the Samsung Medical Center. Patients were excluded from the final analysis if: (1) re-irradiation was performed for local recurrence (*n* = 28), (2) PRT was not completed (*n* = 5), or (3) follow-up data were unavailable (*n* = 3). Among 5 patients who did not complete PRT, 3 patients refused to receive any further treatments and 2 patients showed rapidly progressive systemic disease resulting in early administration of systemic treatments. A total of 133 patients were identified in the final analysis. The requirement for informed consent was waived owing to the retrospective nature of the study.

### Definition of disease status and burden at PRT

First, we categorized the disease extent based on the affected organs (lymph node, liver, lung, bone, or brain) at diagnosis and PRT. Based on international consensus guidelines for advanced breast cancer statements, oligometastatic disease was defined as ≤ 5 lesions involved at the time of PRT [22]. In addition, stage IV disease at PRT was categorized into three groups: de novo stage IV, progressive stage IV (progressive disease after treatment for de novo stage IV disease), and recurrent IV groups (recurrent disease with distant metastasis after treatment for the localized disease). Lastly, we classified patients into groups according to disease burden outside the breast at the time of breast PRT based on the RECIST criteria: stable disease, progressive disease, and mixed responses. In the assessment of the disease burden outside the breast, patients with de novo stage IV disease were categorized as having stable disease.

### Radiation treatment

With an interval of 12.3 months (interquartile range [IQR] 5.5–27.5) from the diagnosis of metastatic disease, patients were referred for PRT due to the following reasons: (1) relief of symptoms (e.g., bleeding, discharge, or severe pain) (*n* = 51, 38.3%), and (2) treatment of radiologic progression without symptoms (*n* = 82, 61.7%). When summarized by treatment year, the number of PRT cases has recently increased (Additional file 1: Figure S1A). There is no statistical significance, but recently, the rate of RT for asymptomatic radiologic progression has increased (Additional file 1: Figure S1B). None of the patients underwent PRT following palliative surgery. Either gross breast tumor with margins (*n* = 108, 81.2%) or whole breast/chest wall (*n* = 25, 18.8%) were treated for PRT planning. The gross lesion was defined as gross tumor volume, and the median gross tumor volume was 613.5 cm^3^ (IQR, 313.0–973.6). Overall, a total dose of 45.0 Gy (IQR, 45.0–50.0) was prescribed; an equivalent dose in 2 Gy fractions (EQD2, assuming α/β as 3.5) was 53.2 Gy (IQR, 53.2–63.8). Details of dose scheme are summarized in Additional file 1: Table S1. A total dose of 45 Gy in 15 fractions was the most frequent dose scheme (*n* = 56, 42.1%) followed by 54 Gy in 18 fractions (*n* = 14, 10.5%), 48 Gy in 10 fractions (*n* = 10, 7.5%), and 60 Gy in 15 fractions (n = 10, 7.5%). Most patients were treated with three-dimensional conformal RT (*n* = 74, 55.6%), followed by intensity-modulated RT (*n* = 58, 43.6%) and proton beam therapy (*n* = 1, 0.8%). Regarding systemic treatment, 46 patients (34.6%) received PRT between cytotoxic chemotherapy cycles or concurrent cytotoxic chemotherapy, and 27 (20.3%) and 10 (7.5%) patients received concurrent endocrine or anti-HER2 targeted therapy, respectively. Fifty patients (37.6%) did not receive systemic treatment within one month before and after PRT.

### Statistical analysis

Response after PRT was assessed based on RECIST. Local control (LC) was calculated from the date of PRT to the date of local failure. Overall survival (OS) was calculated from the date of PRT to death from any cause or last follow-up. LC, and OS rates were estimated using the Kaplan–Meier method. The Cox proportional hazards model was used for multivariable analysis of factors affecting LC, and OS; only factors with statistical significance in the univariable analysis were included. The optimal cut-off value for gross tumor volume and EQD2 was evaluated using the R package “MaxStat," which iteratively tests all possible cut-off points to find the one achieving the maximum rank statistic [23]. A two-sided *P*-value of < 0.05 was considered statistically significant. All statistical analyses were performed using R software (version 4.2.2; R Foundation for Statistical Computing, Vienna, Austria).

## Result

### Patient characteristics

The patient and tumor characteristics are summarized in Table 1. The median age at breast PRT was 51 years (IQR, 45–57), and most patients had bone metastases (*n* = 65, 48.9%). The median two lines (IQR, 1–4) of systemic therapy were administered before PRT. Most of the patients (*n* = 120, 90.2%) had polymetastatic disease at the time of breast PRT. Specifically, 65 patients (48.9%) had single-organ metastatic disease, followed by 38 (28.6%) and 30 (22.6%) patients with 2 or 3–4 organ involvements, respectively. Regarding disease status at PRT, more than half (*n* = 76, 57.1%) of the patients had progressive stage IV disease, whereas 30 patients (22.6%) and 27 patients (20.3%) received PRT at de novo stage IV and recurrent stage IV disease, respectively. In addition, 104 patients (78.2%) had stable disease outside the breast, whereas 17 (12.8%) and 12 (9.0%) patients had progressive disease and mixed responses, respectively at sites other than the breast.

**Table 1:** Patient, tumor, and treatment characteristics

		N (%) or Median [IQR]
Age		51.0 [45.0–57.0]
Subtype of breast tumor	HR positive/HER2 negative	48 (36.1)
	HR positive/HER2 positive	19 (14.3)
	HR negative/HER2 positive	19 (14.3)
	TN	47 (35.3)
Disease extent at initial breast cancer diagnosis		
	Lymph node	49 (36.8)
	Liver	23 (17.3)
	Lung	52 (39.1)
	Bone	65 (48.9)
	Brain	1 (0.8)
Treatment era	2010–2015	52 (39.1)
	2016–2021	81 (60.9)
Numbers of previous systemic treatments (lines)		2 [1–4]
Systemic treatment-free interval (months)		0.8 [0.0–1.2]
Number of metastatic lesions	Oligometastasis (≤ 5 lesions)	13 (9.8)
	Polymetastasis (> 5 lesions)	120 (90.2)
Number of organ systems metastases	1	65 (48.8)
	2	38 (28.6)
	3–4	30 (22.6)
Disease extent at breast palliative RT	Lymph node	61 (45.9)
	Liver	26 (19.5)
	Lung	59 (44.4)
	Bone	72 (54.1)
	Brain	5 (3.8)
Status	De novo stage IV	30 (22.6)
	Progressive stage IV	76 (57.1)
	Recurrent stage IV	27 (20.3)
Disease burden outside the breast	Stable disease	104 (78.2)
	Progressive disease	17 (12.8)
	Mixed response	12 (9.0)

*HR* Hormone receptor, *HER2* Human epidermal growth factor receptor 2, *TN* Triple negative, *RT* Radiation therapy

### Clinical outcomes

**Fig. 1:**
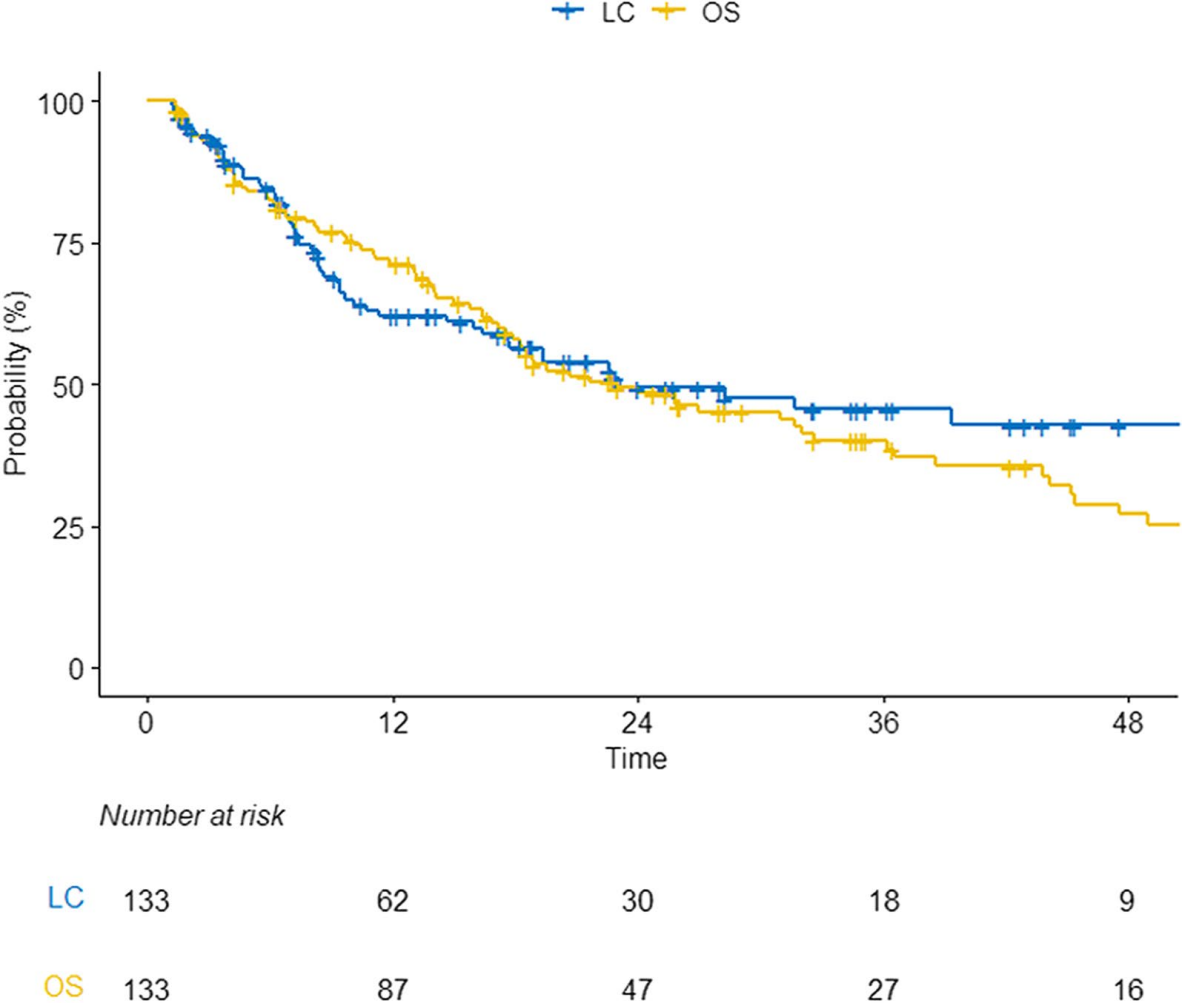
Clinical outcomes in the study cohort *LC* Local control, *OS* Overall survival

The overall response rate for breast tumors following PRT was 74.4%; complete, partial response, stable disease, and progressive disease was observed in 9.0%, 65.4%, 18.8%, and 6.8%, respectively. Among 51 patients (38.3%) with symptomatic disease, 5 (9.8%) and 34 (66.7%) patients experienced disappeared and decreased pain/bleeding, respectively; 12 (23.5%) showed no response after PRT. In addition, among 82 (63.7%) patients with asymptomatic radiologic progression, 7 (8.5%) and 53 (64.6%) showed complete remission and partial response, respectively, whereas 22 (26.8%) showed stable or progressive disease. There were no grade 3 or more toxicities related to PRT. During a median follow-up of 17.2 months (IQR, 7.3–32.0), the 2-year LC and OS rates were 49.4%, and 48.3%, respectively (Fig. 1).

Patients treated with ≤ 2 lines of systemic therapy (*n* = 72, 54.1%), those with de novo stage IV disease (*n* = 30, 22.6%), and those with stable disease outside the breast (*n* = 104, 78.2%) showed favorable LC and OS outcomes (Table 2, Additional file 1: Table S2). In addition, breast PRT with ≥ 63 Gy (EQD2, n = 61, 45.9%) showed a statistically significant benefit in LC than that with < 63 Gy (2-year LC, 67.7% *vs.* 42.9%, *P* = 0.010). Additionally, either hormone receptor positive tumor treated with endocrine therapy or HER2 positive tumor treated with anti-HER2 therapy showed superior OS outcomes than others (Table 2, Additional file 1: Table S2).

**Table 2:** Local control (LC) outcomes according to clinical factors

		N (%)	2-year LC	*P*-value
Entire			49.4%	
Age	< 45 years	33 (24.8)	48.7%	0.830
	≥ 45 years	100 (75.2)	50.0%	
Subtype	HR positive/HER2 negative	48 (36.1)	56.0%	0.210
	HR positive/HER2 positive	19 (14.3)	34.3%	
	HR negative/HER2 positive	19 (14.3)	30.0%	
	TN	47 (35.3)	57.1%	
Treatment era	2010–2015	52 (39.1)	42.6%	0.087
	2016–2021	81 (60.9)	55.8%	
Previous systemic treatments	≤ 2 lines	72 (54.1)	62.7%	< .001
	> 2 lines	61 (45.9)	27.6%	
Interval from diagnosis to PRT	≤ 1 year	65 (48.9)	58.5%	0.053
	> 1 year	68 (51.1)	38.9%	
			No *vs.* Yes	
Disease extent at PRT	LN	61 (45.9)	55.7% *vs.* 42.5%	0.110
	Liver	26 (19.5)	50.4% *vs.* 44.1%	0.260
	Lung	59 (44.4)	46.2% *vs.* 54.9%	0.810
	Bone	72 (54.1)	52.1% *vs.* 48.5%	0.860
	Brain	5 (3.8)	50.9% *vs.* 0.0%	< .001
Number of metastases	Oligometastasis (≤ 5 lesions)	13 (9.8)	66.7%	0.330
	Polymetastasis (> 5 lesions)	120 (90.2)	47.6%	
Status	D*e novo* stage IV	30 (22.6)	64.2%	0.076
	Progressive stage IV	76 (57.1)	46.1%	
	Recurrent stage IV	27 (20.3)	45.1%	
Disease burden (outside breast)	SD	104 (78.2)	54.1%	0.003
	PD or mixed response	29 (21.8)	24.4%	
Reason for breast PRT	Symptom (bleeding, pain)	51 (38.3)	62.3%	0.180
	Radiologic progression	82 (61.7)	42.1%	
Concurrent treatment	No	50 (37.6)	54.3%	0.360
	Endocrine therapy	27 (20.3)	59.2%	
	Anti-HER2 therapy	10 (7.5)	50.0%	
	Cytotoxic chemotherapy	46 (34.6)	34.8%	
Gross tumor volume	< 1260 cm^3^	107 (80.5)	50.2%	0.110
	≥ 1260 cm^3^	26 (19.5)	46.2%	
PRT dose (EQD2)	< 63 Gy	72 (54.1)	42.9%	0.010
	≥ 63 Gy	61 (45.9)	67.7%	
PRT modality	3D-CRT	74 (55.6)	44.5%	0.330
	IMRT/Proton	59 (44.4)	56.5%	

*HR* Hormone receptor, *HER2* Human epidermal growth factor receptor 2, *TN* Triple-negative, *LN* Lymph node, *SD* Stable disease, *PD* Progressive disease, *PRT* Palliative radiation therapy, *EQD2* Equivalent dose in 2 Gy fractions (α/β = 3.5), 3D-CRT, 3 dimensional-conformal radiation therapy; IMRT, intensity-modulated radiation therapy

After multivariable analysis,  > 2 lines of previous systemic treatments (hazard ratio [HR], 2.84; 95% confidence interval [CI] 1.50–5.36, *P* = 0.001), progressive disease or mixed responses at other sites (HR 2.20, 95% CI 1.15–4.20, *P* = 0.009), and breast PRT < 63 Gy (HR 0.42, 95% CI 0.20–0.88, *P* = 0.017) were associated with inferior LC (Table 3).

**Table 3:** Prognostic factors for local control

Local control		Univariable analysis			Multivariable analysis		
		HR	95% CI	*P*-value	HR	95% CI	*P*-value
Age	(< 45 *vs.* ≥ 45 years)	1.07	0.58–1.96	0.832			
Subtype	(HR positive *vs.* HER2 positive)	1.79	0.95–3.37	0.070			
	(HR positive *vs.* TN)	1.08	0.56–2.07	0.825			
Treatment year	(continuous)	0.94	0.88–1.02	0.126			
Treatment era	(2010–2015 vs. 2016–2021)	0.63	0.37–1.07	0.089			
Previous systemic treatment	(≤ 2 *vs.* > 2 lines)	2.81	1.64–4.81	< .001	2.84	1.50–5.36	0.001
Interval from diagnosis to PRT	(≤ 1 *vs.* > 1 year)	1.68	0.99–2.85	0.055			
Number of metastatic lesions	(≤ 5 *vs.* > 5 lesions)	1.64	0.59–4.54	0.340			
Disease status	(de novo stage IV *vs.* recurrent stage IV)	1.83	0.90–3.70	0.094	0.80	0.34–1.89	0.612
	(de novo stage IV *vs.* progressive Stage IV)	2.64	1.11–6.31	0.028	1.55	0.60–4.03	0.366
Disease burden outside breast	(SD *vs.* PD/mixed response)	2.47	1.33–4.58	0.004	2.20	1.15–4.20	0.009
Reason for breast PRT	(Symptomatic *vs.* asymptomatic)	1.47	0.83–2.61	0.184			
Concurrent treatment	(No *vs.* hormonal therapy)	0.71	0.35–1.46	0.354			
	(No *vs.* anti-HER2 therapy)	0.83	0.31–2.22	0.712			
	(No *vs.* cytotoxic chemotherapy)	1.35	0.71–2.54	0.357			
Gross tumor volume	(Continuous, per 100 cc)	1.01	0.97–1.05	0.606			
Gross tumor volume	(< 1260 vs. ≥ 1260 cm^3^)	1.63	0.89–2.98	0.114	1.62	0.86–3.05	0.136
PRT dose, EQD2	(< 63 *vs.* ≥ 63 Gy)	0.40	0.20–0.82	0.012	0.42	0.20–0.88	0.017
PRT modality	(3D-CRT *vs.* IMRT/proton therapy)	0.76	0.44–1.31	0.332			

*HR* Hormone receptor, *HER2* Human epidermal growth factor receptor 2, *TN* Triple-negative, *SD* Stable disease, *PD* Progressive disease, *PRT* Palliative radiation therapy, *EQD2* Equivalent dose in 2 Gy fractions (α/β = 3.5); 3D-CRT, 3 dimensional-conformal radiation therapy, *IMRT* Intensity-modulated radiation therapy

### Risk group stratification

**Fig. 2:**
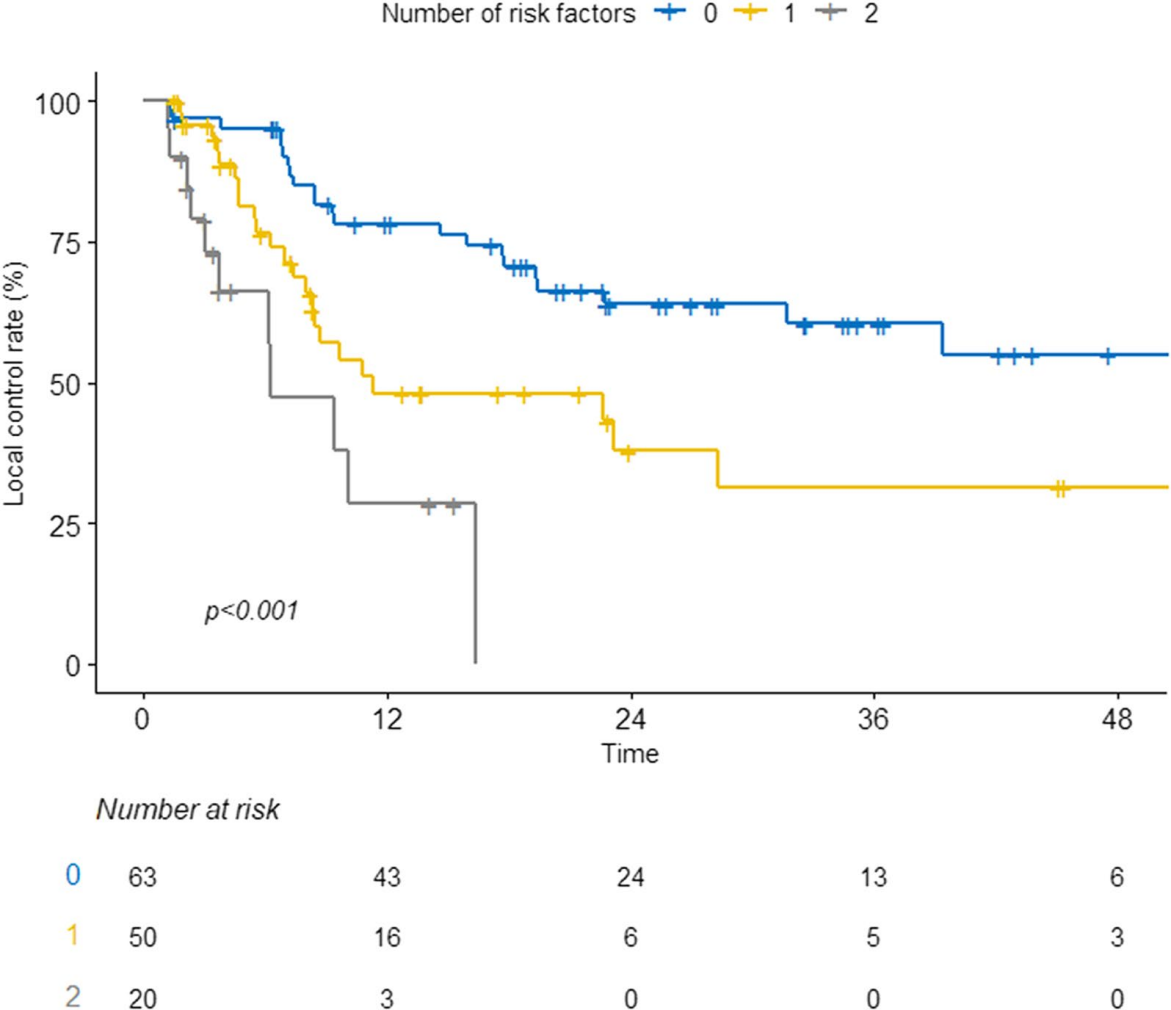
Local control stratified by number of risk factors. *Risk factors included previous systemic treatments of more than two lines, progressive disease, and mixed responses observed in disease burdens outside the breast. *LC* Local control, *DMPFS* Distant metastasis progression-free survival; OS, overall survival

Considering the results multivariable analysis, we stratified patients into three groups based on risk factors of > 2 lines of previous systemic therapy and progressive disease/mixed responses outside the breast: group 1 (no risk factors, *n* = 63), group 2 (one risk factor, *n* = 50), and group 3 (two risk factors, *n* = 20). A significant difference in clinical outcomes was observed between the risk groups (Fig. 2). Specifically, the 2-year LC rates for groups 1, 2, and 3 were 63.9%, 43.2%, and 0%, respectively (*P* < 0.001, Fig. 2). In addition, the 2-year OS rates were 72.8%, 35.8%, and 6.8% (Additional file 1: Figure S2) for groups 1, 2, and 3, respectively.

**Fig. 3:**
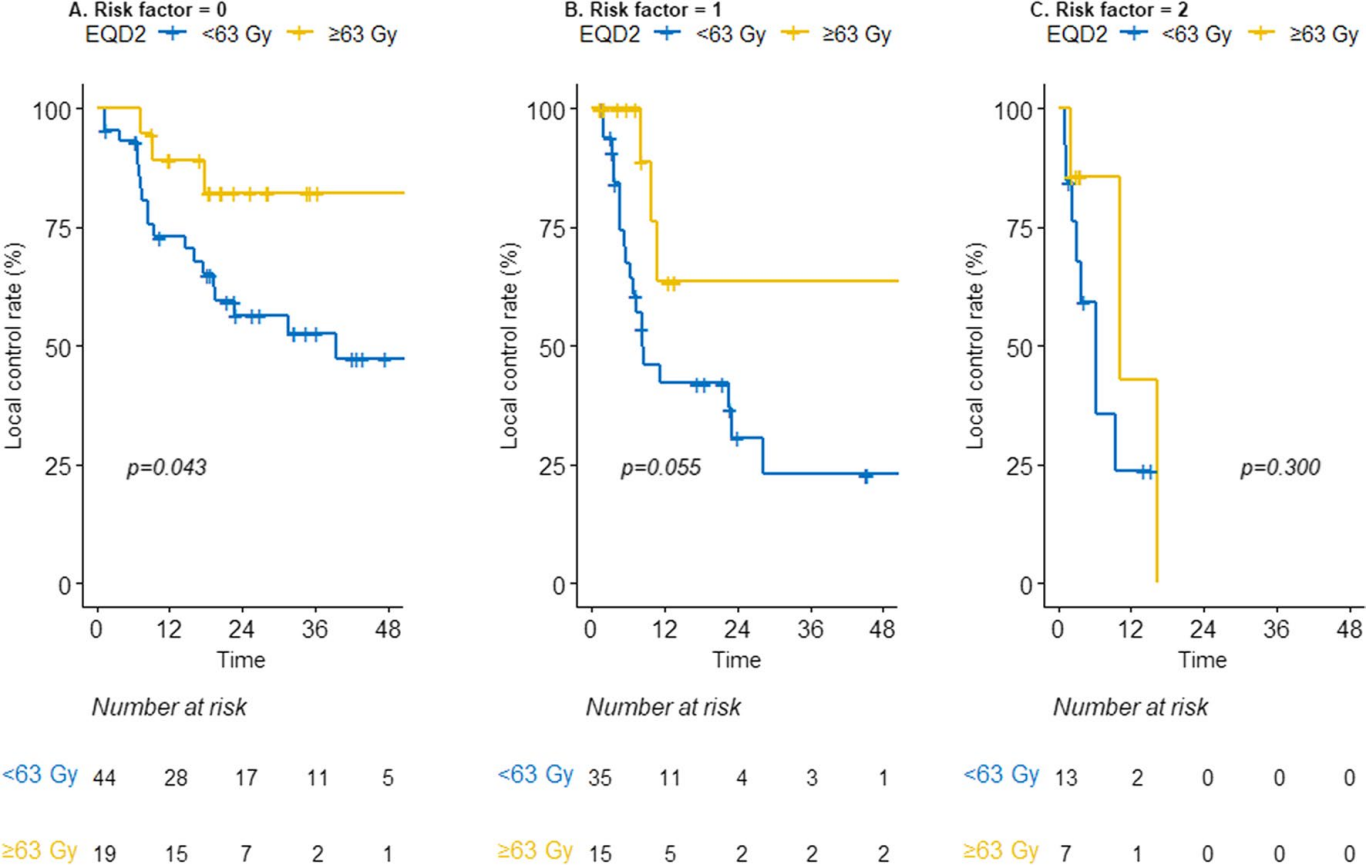
Local control in risk groups stratified by radiation dose: Low-risk **A**, Intermediate-risk** B**, and High-risk** C**. *EQD2* Equivalent dose in 2 Gy fractions (α/β = 3.5)

The impact of high-dose PRT (≥ 63 Gy) was further evaluated in subgroup analyses based on risk factors. In these analyses, ≥ 63 Gy showed a statistically significant benefit in LC (Fig. 3A) for group 1. In addition, high-dose PRT was related to borderline significance in LC in group 2 (2-year rate, 63.5% *vs.* 30.7%, *P* = 0.055, Fig. 3B). There was no improvement after ≥ 63 Gy in LC in group 3 (Fig. 3C).

## Discussion

In this retrospective study, we observed clinical outcomes following PRT for locally uncontrolled breast cancer. Despite the heterogeneity of the patients in the analyzed disease subtypes, the extent of the metastasis, previous treatments received, overall disease burden, treatment histories with less than three lines of previous systemic treatments and stable disease statuses outside the breast were suggested as low-risk groups presenting favorable clinical outcomes. Additionally, the effect of PRT ≥ 63 Gy on LC was apparent in the low-risk group, thereby addressing the need for risk-adjusted PRT in stage IV patients with locally unresectable breast cancer.

Various modalities for local treatment can be employed to control breast tumors and alleviate local symptoms in patients with stage IV breast cancer. Breast PRT or surgical resection, with or without postoperative RT, can be adopted as for local therapeutic modalities. Based on the theory of primary cancer as a persistent source of circulating tumor cells, it is thought that PRT to the primary tumor can eradicate the circulating tumor cells, potentially leading to the control of metastatic disease outside the breast in selective patients [23, 24]. Several retrospective studies have provided data suggesting the potential role of PRT for the primary tumor in de novo stage IV breast cancer [9–11]. Scodan et al. compared 320 patients treated with local treatment, mainly with PRT (only 9.4% of patients received primary tumor resection alone), with 261 patients without local treatment and de novo stage IV disease [9]. They reported superior 3-year OS rates of 43.4% after local treatment compared to 26.7% after no local treatment (*P* < 0.001). In addition, Bourgier et al. reviewed 308 patients with stage IV disease, showing that PRT could provide long-term LC in 85% of patients, with better OS outcomes than surgical resection [10]. Further, a French multicenter cohort study of 1965 patients demonstrated that PRT (HR 0.63, 95%CI 0.49–0.80) was associated with a comparable OS benefit to primary tumor resection followed by postoperative RT (HR 0.61, 95% CI 0.47–0.78) [11]. In their study, although the PRT alone group had larger tumors and greater tumor burdens than the surgery alone or surgery followed by RT groups, the PRT group demonstrated better OS and progression-free survival outcomes than the surgery alone group.

As demonstrated above, previous studies have presented outcomes following PRT, mainly focused on patients with de novo stage IV breast cancer. Nonetheless, there is a paucity of data concerning the role of breast PRT in cases of recurrent or progressive stage IV disease. In one study, Mauro et al. evaluated the role of PRT at the primary site in 125 patients with stage IV disease [12]. They included 94 patients (76.0%) who were previously treated with systemic treatment before PRT. In their study, the 3-year OS and local progression-free survival rates were 21.2% and 67.3%, respectively. Consistent with our results, they also found that more than three lines of previous systemic treatment adversely affected local progression-free survival outcomes compared with 1–3 lines (3-year rates, 54.7% *vs.* 68.9%, *P* = 0.048). Based on the results of our study, potential candidates for PRT with curative intent could be expanded to include patients treated with 1–2 lines of systemic treatment. Adopting PRT in not only de novo stage IV patients but also in the currently suggested low-risk group could result in a favorable median survival of 43.8 months, with a 3-year estimated OS of 59.1%.

Meanwhile, surgical resection of the breast tumor, with or without postoperative RT, exhibited favorable LC in stage IV breast cancer [13–19, 25]. Studies focusing on de novo stage IV breast cancer have reported that administering local treatments, such as breast tumor resection followed by postoperative RT, was associated favorable LC[25]. However, invasive surgical procedures may induce surgical dissemination through the adhesion of circulating tumor cells to the vascular endothelium of target organs, immunosuppression, and inflammatory cascades resulting in the growth of metastatic tumors following surgical removal [26]. In this aspect, a recent practical algorithm suggested careful patient selection for intensive local treatment, including both surgery and RT; specifically, patients with non-triple negative breast cancer with controlled de novo stage IV disease [27]. The main strength of breast PRT could be the avoidance of invasive procedures, which could delay systemic therapy and lead to complications due to unhealed wounds. Therefore, breast PRT could be a reasonable treatment option other than surgical resection, as locally uncontrolled primary tumors can seriously affect patients’ quality of life. Indeed, a prospective study by Nakamura et al. showed the efficacy of PRT in relieving bleeding or discharge in locally aggressive tumors with skin invasion [28]. Collectively, we expect that timely breast PRT can allow long-term local and distant disease control in selected stage IV patients.

Regarding the prescription dose, limited data is available to suggest optimal PRT schedules [12, 28–30]. Vempati et al. reported that no clinical response was observed in patients treated with PRT < 30 Gy (EQD2), whereas 69% of patients treated with PRT ≥ 30 Gy (EQD2) showed meaningful clinical improvement in ulcerative breast lesions [29]. Although the 21 patients mostly treated with 36 Gy in 12 fractions reported by Nakamura et al. showed symptom relief at 3 months, re-progression of symptoms occurred at 6 months [28]. This suggests that optimization of PRT schedules is necessary to achieve long-term control. In another study, Choi et al. observed a trend toward improved symptom relief after ≥ 80 Gy (biologically effective dose, assuming α/β as 4.0) compared with < 80 Gy (HR 0.70, *P* = 0.06) in locally incurable inflammatory breast cancer[30] (80 Gy of the biologically effective dose could be translated into 53 Gy in EQD2). Mauro et al. also demonstrated that PRT with > 30 Gy was associated with superior local progression-free survival compared to ≤ 30 Gy after adjusting for clinical factors. In their study, the 3-year rates of local progression-free survival were 74.9% and 49.3% following > 30 Gy and ≤ 30 Gy, respectively (*P* = 0.028) [12]. Consistent with previous reports, we found a significant and marginal improvement in LC after PRT ≥ 63 Gy for patients with low-risk and intermediate-risk group, respectively.

The interpretation of the current study has several limitations owing to its retrospective nature. First, the heterogeneity and retrospective nature of the current cohort hindered a comprehensive analysis to assess the beneficial impact of PRT. Although uniform EQD2 was calculated for analysis, a large variety of dose scheme (Additional file 1: Table S1) was adopted. In addition, the small number of patients in each subgroup could dilute the potential benefit of ≥ 63 Gy. Also, as we only collected clinicopathological factors through chart reviews, the biological backgrounds of patients (e.g. somatic gene mutation, PD-L1 status, cell-free DNA, etc.) could not be identified, which could play a key role in those with high tumor burdens. Further studies are warranted to verify the potential role of PRT with specific dose regimen in locally unresectable disease and to identify the optimal candidates for intensive PRT. Also, the current cut-off of 63 Gy EQD2 should be validated in other cohorts since only 28% of patients received PRT ≥ 63 Gy.

In conclusion, breast PRT could provide favorable LC in stage IV patients with locally uncontrolled disease, regardless of their overall disease burdens or previous treatments. Patients with stable disease outside the breast and who were treated with less than three lines of systemic treatment showed favorable outcomes. As a hypothesis-generating study, PRT with ≥ 63 Gy has a potential of long-term disease control in these low-risk patients. Based on our results, a total dose of 44–45 Gy in 10 fractions (63–66 Gy in EQD2), 36 Gy in 6 fractions (62 Gy in EQD2), or 35 Gy in 5 fractions (67 Gy in EQD2) can be recommended for PRT in low- or intermediate-risk groups for long-standing LC. Given the dismal outcomes observed in the high-risk group (group 3), it is postulated that a short course of RT, delivering around 30 Gy, may be necessary to achieve symptoms relief. Further confirmatory, prospective, randomized controlled trial is crucial to validate these results.

### Supplementary Information

**Additional file 1:** Supplementary Data.

## Data Availability

The datasets generated and analyzed during the current study are not publicly available due to institutional data protection law and confidentiality of patient data but are available from the corresponding author on reasonable request in person.
