# Supplementary material for: Impact of high dose radiotherapy for breast tumor in locoregionally uncontrolled stage IV breast cancer: a need for a risk-stratified approach
Source: Radiat Oncol. 2023 Oct 11;18:168. doi: 10.1186/s13014-023-02357-7 (PMC10566115; doi:10.1186/s13014-023-02357-7)

**Supplementary Table 1. Details of palliative radiotherapy**

|  |  | **N (%)** |
| --- | --- | --- |
| Total dose / number of fractions (EQD2) | 45 Gy / 15 fractions (53.2 Gy) | 56 (42.1) |
|  | 54 Gy / 18 fractions (63.8 Gy) | 14 (10.5) |
|  | 48 Gy / 10 fractions (72.4 Gy) | 10 (7.5) |
|  | 60 Gy / 15 fractions (81.8 Gy) | 10 (7.5) |
|  | 40 Gy / 10 fractions (54.5 Gy) | 9 (6.8) |
|  | 50 Gy / 20 fractions (54.5 Gy) | 5 (3.8) |
|  | 39 Gy / 13 fractions (46.1 Gy) | 4 (3.0) |
|  | 50 Gy / 10 fractions (77.3 Gy) | 3 (2.3) |
|  | 50 Gy / 25 fractions (50.0 Gy) | 3 (2.3) |
|  | 42 Gy / 14 fractions (49.6 Gy) | 3 (2.3) |
|  | 30 Gy / 10 fractions (35.5 Gy) | 3 (2.3) |
|  | 52 Gy/ 13 fractions (70.9 Gy) | 3 (2.3) |
|  | 35 Gy / 5 fractions (66.8 Gy) | 2 (1.5) |
|  | 36 Gy / 10 fractions (46.5 Gy) | 2 (1.5) |
|  | 40 Gy / 15 fractions (43.6 Gy) | 2 (1.5) |
|  | 75 Gy / 30 fractions (81.8 Gy) | 2 (1.5) |
|  | 32 Gy / 4 fractions (66.9 Gy) | 1 (0.8) |
|  | 24 Gy / 6 fractions (32.7 Gy) | 1 (0.8) |

**Abbreviations:** EQD2, equivalent dose in 2 Gy fractions (α/β=3.5).

**Supplementary Table 2. Overall survival (OS) according to clinical factors**

|  |  | N (%) | 2-year OS | *P*-value |
| --- | --- | --- | --- | --- |
| Entire |  |  | 48.3% |  |
| Age | <45 years | 33 (24.8) | 55.0% | 0.830 |
|  | ≥45 years | 100 (75.2) |  |  |
| Subtype | HR positive/HER2 negative | 48 (36.1) | 68.5% | 0.064 |
|  | HR positive/HER2 positive | 19 (14.3) | 39.7% |  |
|  | HR negative/HER2 positive | 19 (14.3) | 31.6% |  |
|  | TN | 47 (35.3) | 39.0% |  |
| Treatment era | 2010-2015 | 52 (39.1) | 40.4% | 0.039 |
|  | 2016-2021 | 81 (60.9) | 55.4% |  |
| Previous systemic treatments | ≤2 lines | 72 (54.1) | 69.9% | <.001 |
|  | >2 lines | 61 (45.9) | 23.3% |  |
| Interval from diagnosis to PRT | ≤1 year | 65 (48.9) | 58.6% | 0.013 |
|  | >1 year | 68 (51.1) | 38.5% |  |
|  |  |  | No *vs.* Yes |  |
| Disease extent at PRT | LN | 61 (45.9) | 52.5% *vs.* 44.4% | 0.200 |
|  | Liver | 26 (19.5) | 48.3% *vs.* 49.9% | 0.640 |
|  | Lung | 59 (44.4) | 53.3% *vs.* 42.4% | 0.250 |
|  | Bone | 72 (54.1) | 44.6% *vs.* 51.5% | 0.680 |
|  | Brain | 5 (3.8) | 50.3% *vs.* 0.0% | <.001 |
| Number of metastases | Oligometastasis (≤5 lesions) | 13 (9.8) | 63.9% | 0.042 |
|  | Polymetastasis (>5 lesions) | 120 (90.2) | 46.6% |  |
| Status | D*e novo* stage IV | 30 (22.6) | 85.2% | <.001 |
|  | Progressive stage IV | 76 (57.1) | 43.5% |  |
|  | Recurrent stage IV | 27 (20.3) | 12.0% |  |
| Disease burden (outside breast) | SD | 104 (78.2) | 56.1% | <.001 |
|  | PD or mixed response | 29 (21.8) | 17.7% |  |
| Reason for breast PRT | Symptom (bleeding, pain) | 51 (38.3) | 44.2% | 0.190 |
|  | Radiologic progression | 82 (61.7) | 51.1% |  |
| Concurrent treatment | No | 50 (37.6) | 36.6% | <.001 |
|  | Endocrine therapy | 27 (20.3) | 84.1% |  |
|  | Anti-HER2 therapy | 10 (7.5) | 90.0% |  |
|  | Cytotoxic chemotherapy | 46 (34.6) | 25.4% |  |
| Gross tumor volume | <1260 cm^3^ | 107 (80.5) | 49.0% | 0.930 |
|  | ≥1260 cm^3^ | 26 (19.5) | 45.7% |  |
| PRT dose (EQD2) | <63 Gy | 72 (54.1) | 46.6% | 0.410 |
|  | ≥63 Gy | 61 (45.9) | 53.1% |  |
| PRT modality | 3D-CRT | 74 (55.6) | 44.4% | 0.240 |
|  | IMRT/Proton | 59 (44.4) | 54.1% |  |

*Abbreviations*: HR, hormone receptor; HER2, human epidermal growth factor receptor 2; TN, triple-negative; LN, lymph node; SD, stable disease; PD, progressive disease; PRT, palliative radiation therapy; EQD2, equivalent dose in 2 Gy fractions (α/β=3.5); 3D-CRT, 3 dimensional-conformal radiation therapy; IMRT, intensity-modulated radiation therapy.

**Supplementary Figure 1. Number of patients (A) and reason for radiotherapy (B) according to the treatment year**

**Supplementary Figure 2. Overall survival outcomes according to number of risk factors.**


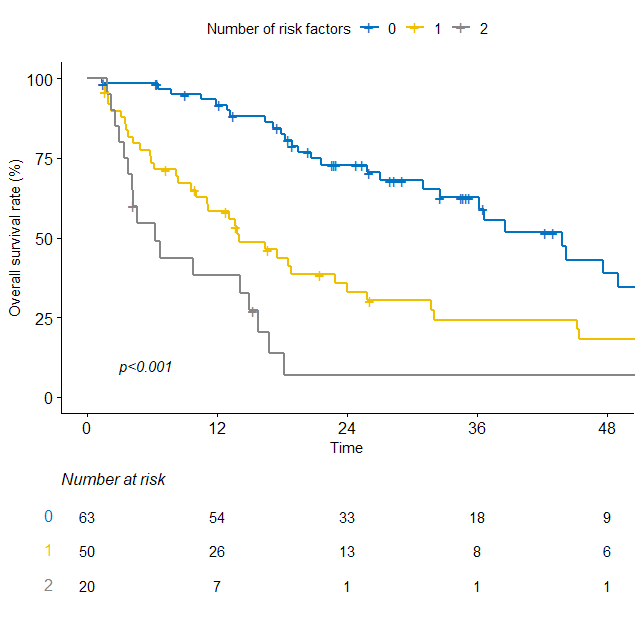

Supplement: Supplementary file 1 — Additional file 1: Supplementary Data. [file 13014_2023_2357_MOESM1_ESM.docx]
